# Prognostic Risk Stratification and End-of-Life Care Outcomes in Patients With Metastatic Melanoma Treated With Immune Checkpoint Inhibitors

**DOI:** 10.1093/oncolo/oyad219

**Published:** 2023-08-05

**Authors:** Robert N Grad, Seungyeon Jung, Fei Ye, Lili Sun, Douglas B Johnson, Rajiv Agarwal

**Affiliations:** Department of Medicine, Vanderbilt University Medical Center, Nashville, TN, USA; Department of Medicine, Vanderbilt University School of Medicine, Nashville, TN, USA; Department of Biostatistics, Vanderbilt University Medical Center, Nashville, TN, USA; Department of Biostatistics, Vanderbilt University Medical Center, Nashville, TN, USA; Department of Medicine, Vanderbilt University Medical Center, Nashville, TN, USA; Department of Medicine, Vanderbilt University Medical Center, Nashville, TN, USA

**Keywords:** immune checkpoint inhibition, prognostication, melanoma, end-of-life care, hospice

## Abstract

**Introduction:**

The emergence of immune checkpoint inhibitors (ICIs) has improved survival outcomes in patients with metastatic melanoma, while potentially increasing the use of systemic therapy near the end of life (EOL). Yet, less is known on how to facilitate treatment decision making and identify patients who might benefit from early palliative care comanagement.

**Materials and Methods:**

We determined baseline clinical and laboratory factors that are associated with poor prognosis for patients with advanced melanoma treated with ICIs. We subsequently identified prognostic subgroups to evaluate association with EOL outcomes and determine if EOL care varied across prognostic strata.

**Results:**

Our cohort included 398 patients with metastatic melanoma treated with ICIs. Factors associated with overall survival (OS) included: lactate dehydrogenase, neutrophil/lymphocyte ratio, performance status, prior therapies, liver metastases, and lung metastases. Patients were stratified by risk of death using risk scores developed from multivariable analyses. A total of 205 patients died: 45/133 (34%) low-risk, 63/133 (47%) medium-risk, and 97/132 (73%) of high-risk patients. Among those who died, higher risk patients were more likely to receive ICIs within 14, 30, and 90 days of death. We found no association between risk group and hospice referrals or location of death.

**Conclusion:**

Patients with metastatic melanoma at highest risk of death as defined by our model were more likely than lower-risk patients to receive ICIs near the EOL. Prognostic risk stratification may guide early palliative care interventions to appropriately utilize ICIs and optimize EOL care.

## Teaser

Results of this study show that identifying prognostic subgroups of patients with advanced melanoma could be used to optimize end-of-life care and guide earlier and personalized palliative care interventions.

**Implications for Practice**

This study demonstrates that identifying prognostic subgroups of patients with advanced melanoma could be used to optimize end-of-life care and guide earlier and personalized palliative care interventions. By utilizing routinely available, disease-specific clinical and laboratory values to ascertain prognostic risk, oncology care teams can help patients and families navigate illness understanding and prognostic awareness, can limit ineffective use of immune checkpoint inhibitors, and can facilitate care transitions while honoring personal values at the end of life.

## Introduction

The development of immune checkpoint inhibitors (ICIs) has drastically changed the therapeutic landscape of melanoma. Prior to their advent, patients with metastatic melanoma had a very poor prognosis with a 1-year survival rate of only 25.5%, and median overall survival (OS) of only 5-11 months.^1-3^ Patients with metastatic melanoma treated with programmed death-1 (PD-1) inhibitors (nivolumab, pembrolizumab) have median progression free survival (PFS) of 7-12 months, 1-year survival rate of 73%-74%, 3-year survival rate of 51%-52%, and median OS of 37-39 months.^3-7^ Combination therapy with PD-1 and cytotoxic T-lymphocyte-associated protein 4 (CTLA-4) inhibitors have demonstrated even greater survival benefits with median PFS of 10-12 months, 1-year survival rates of 80%-89%, and 3-year survival rates of 58%-73%.^3-6^ In addition, though ICI treatment may be complicated by immune-related adverse events (irAEs), ICIs are not generally associated with common side effects of cytotoxic chemotherapy, such as nausea, vomiting, neuropathy, alopecia, and myelosuppression.

Notably, due to improved survival outcomes, the potential for durable responses, and acceptable toxicity profile of ICIs, there has been an increase in the use of systemic therapy at the end of life (EOL). One study showed that patients with metastatic melanoma had an increase in the use of systemic therapy within 30 days of death from 33.9% to 43.2%, as the approval of ICIs.^8^ Another study showed that rates of systemic therapy at EOL for patients with metastatic urothelial carcinoma doubled from 17.4% to 34.8%, which was largely accounted for by ICIs.^9^

Emerging data, however, question the utility of ICI treatment in patients with poor prognosis at the end of life. Past studies have shown that patients with advanced NSCLC and urothelial carcinoma with poor performance status treated with ICIs have poor OS outcomes.^10,11^ Other studies have also demonstrated worse outcomes for patients with advanced melanoma with poor prognostic factors (eg, higher metastatic stage, elevated lactate dehydrogenase levels).^12^ Moreover, there is significant variation in practice patterns when initiating new systemic therapy in patients with advanced melanoma near the EOL.^13^ However, to our knowledge, there is limited information to help guide treatment decision making in patients who will not benefit from ICI therapy, and to identify patients who might benefit from upfront palliative care comanagement. Furthermore, it is not clear whether baseline prognostic features drive oncologist behavior (eg, referral to palliative care specialists or engagement in serious illness conversations resulting in earlier recommendation of hospice care).

Therefore, we developed a prognostic model using validated clinical and laboratory factors for patients with advanced melanoma treated with ICIs in order to stratify patients into prognostic subgroups. We subsequently described the association of these risk groups with EOL outcomes to determine if EOL care and practice patterns varied across prognostic strata. Our overall aim was to evaluate if prognostic risk stratification can be helpful to identify a patient population that could benefit from earlier palliative care interventions and help prevent unnecessary and suboptimal treatment for patients who have poorer prognoses.

## Materials and Methods

### Patient Population

After obtaining institutional review board approval, we gathered clinical data from the electronic health record (EHR) for patients at Vanderbilt University Medical Center (VUMC) treated with ICI therapy between October 2009 and March 2020. Patients were included, if they had advanced melanoma and received at least one dose of single-agent PD-1/programmed death ligand-1 (PDL1) inhibitors (nivolumab, pembrolizumab, atezolizumab, avelumab, durvalumab) or a combination of PD-1 and CTLA4 inhibitors (ipilimumab) used in any line of therapy for metastatic disease.

### Study Design

The primary objective of this study was to integrate validated clinical and laboratory factors with prognostic value to stratify patients into high-, intermediate-, and low-risk groups. This grouping would provide prognostic information and ultimately help to assess the association between risk groups and EOL outcomes. Deidentified data were extracted through a review of the EHR. We collected data on patient demographics, baseline laboratory values (ie, serum lactate dehydrogenase (LDH) level and neutrophil/lymphocyte (N/L) ratio), Eastern Cooperative Oncology Group performance status (ECOG PS) as documented by patients’ treating oncologists, clinicopathologic features, prior therapies, treatment responses (OS and PFS), and EOL care. Treatment responses were assessed per Response Evaluation Criteria in Solid Tumors version 1.1 (RECIST 1.1). To assess EOL care, we determined timing of checkpoint inhibitor therapy relative to death, EHR documentation of referral to hospice care, and location of death.

Among patients who received EOL care, some patients, following progression on ICI, received much of their urgent care, inpatient admissions, and palliative care at institutions other than at VUMC (eg, at healthcare facilities closer to their homes). Thus, in EOL analyses, we only included data that were accessible via our institution’s EHR.

### Statistical Analysis

Candidate clinical and laboratory variables were selected a priori based on existing literature.^12^ Numerous studies have already identified variables that have prognostic implications. Age has not been demonstrated to be a significant prognostic factor, whereas LDH, stage, and ECOG PS have been associated with inferior outcomes.^14,15^ A meta-analysis has shown that elevated pretreatment N/L ratio and LDH are associated with worse survival outcomes.^16^ Finally, testing for PD-L1 in melanoma is neither routine nor recommended and, therefore, was not evaluated in this analysis.^17^ A first multivariate regression model included all predictor variables of interest: age, gender, ECOG PS (0/1/2), N/L ratio, LDH, largest tumor size, prior therapies (yes/no), brain metastasis (yes/no), liver metastasis (yes/no), lung metastasis (yes/no), and bone metastasis (yes/no). Backward selection of variables was used to avoid overfitting and to derive a parsimonious model. A final multivariable Cox regression model was fitted to overall survival data with the following variables: ECOG PS (0/1/2), N/L ratio, LDH, prior therapies (yes/no), liver metastasis (yes/no), and lung metastasis (yes/no). The continuous variables, LDH and N/L ratio, were allowed to have a nonlinear relationship with the outcome using a restricted cubic spline function. Missing covariate data were imputed using multiple imputation (R package “Hmisc”). Based on the estimated risk scores from this model, patients were divided into 3 risk groups: low, medium, and high risk. Association between risk group and timing of receipt of checkpoint inhibitor therapy relative to death (14, 30, and 90 days) was evaluated using Pearson’s Chi-squared test. All statistical analyses were performed in R 4.2.1.

## Results

Our cohort included 398 patients (Table 1). One hundred and thirty-eight (35%) were female and 260 (65%) were male. The average age was 60.9 years (range 20-89). Two hundred and sixty-three patients (66%) received anti PD-1 or anti-PDL1 monotherapy while 134 (34%) received combination PD-1 and anti-CTLA-4 therapy. At initiation of therapy, 140 (37%) of the patients had ECOG PS of 0, 205 (54%) had ECOG PS of 1, 35 (9%) had ECOG PS score of 2-3. Two hundred and fourteen (54%) patients had received no prior therapies; 178 (45%) had stage M1a or M1b disease, 133 (33%) had M1c disease, and 85 (21%) had M1d disease. Eighty-five (21%) patients had brain metastases, 105 (26%) had liver metastases 220 (55%) had lung metastases, and 86 (22) had bone metastases. The median LDH level was 209 (range 100-3587), and the median neutrophil/lymphocyte ratio was 3 (range 0.5-58).

**Table 1.** Demographic and baseline characteristics of patients.

Characteristic	No. (%) of patients
Sex	
** Female**	138 (35)
** Male**	260 (65)
Age, mean (range)	60.9 (20-89)
Treatment	
** Anti-PD-1 or anti-PDL1 monotherapy**	263 (66)
** Combination PD-1/CTLA4**	134 (34)
ECOG performance status	
** 0**	140 (37)
** 1**	205 (54)
** 2**	31 (8)
** 3**	4 (1)
Prior therapies	
** No prior therapies**	214 (54)
** At least 1 prior therapy**	181 (46)
Location of metastases	
** Brain**	85 (21)
** Liver**	105 (26)
** Lung**	220 (55)
** Bone**	86 (22)
Metastases stage	
** M1a**	98 (25)
** M1b**	80 (20)
** M1c**	133 (33)
** M1d**	85 (21)
LDH, median (range)	209 (100-3587)
Neutrophil/lymphocyte ratio, median (range)	3 (0.5-58)

Table 1 shows the demographics and baseline characteristics of the patients included in our cohort. Data are presented as *N* (%) unless otherwise specified.

Abbreviations: anti-PD-1: anti-programmed death-1; anti-PDL1: anti-programmed death ligand-1; CTLA4: cytotoxic T-lymphocyte-associated protein 4; ECOG: Eastern Cooperative Oncology Group.

**Figure 1.**
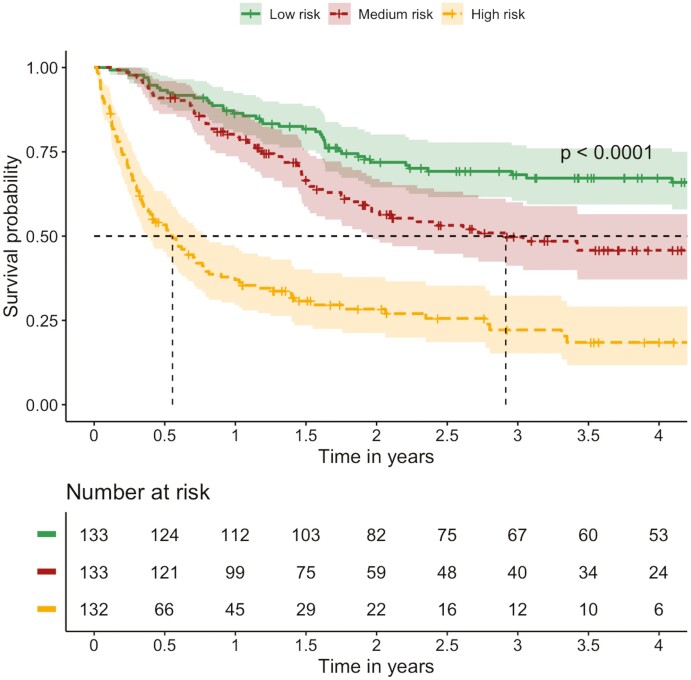
Kaplan-Meier curves among prognostic risk groups. Figure demonstrates the overall survival relative to our prognostic risk stratification. The *Y*-axis demonstrates survival probability, and the *X*-axis demonstrates time in years. Green demonstrates the low-risk group, red demonstrates the medium-risk group, and yellow demonstrates the high-risk group. As expected, patients in the highest risk group had worse overall survival, compared to patients in the lowest risk group.

To assess and confirm the prognostic value of these variables, we performed multivariable analyses to examine their impact on OS. To derive a parsimonious model and avoid overfitting, several variables were associated with OS and selected in our final prognostic model (Table 2). This notably included LDH (interquartile range of HR 1.55, 95%CI, 1.13-2.11, *P* = .012), N/L ratio (interquartile range of HR 1.51, 95%CI, 1.13-2.01, *P* = .076), ECOG PS 1 (HR 1.94, 95%CI, 1.38-2.74, *P* < .001), ECOG PS 2 or greater (HR 4.84, 95%CI, 2.95-7.92, *P* < .001), prior treatment with either immunotherapy or chemotherapy (HR 1.34, 95%CI, 1.00-1.81, *P* = .051), presence of liver metastases (HR 1.41, 95%CI, 1.01-1.99, *P* = .047), and the presence of lung metastases (HR 1.38, 95%CI, 1.02-1.87, *P* = .035). Based on these results, we grouped patients into high, medium, and low risk of death, as demonstrated by Kaplan-Meier curves comparing OS among risk groups in Fig. 1.

**Table 2.** Hazard ratios of variables for determining prognostic scores.

Variable	Hazard ratio for OS (95% CI)	*P*-value
LDH	1.55 (1.13-2.11)	.0125
Neutrophil/lymphocyte ratio	1.51 (1.13-2.01)	.0758
ECOG PS 1	1.94 (1.38-2.74)	.0002
ECOG PS 2 or greater	4.84 (2.95-7.92)	<.0001
Prior therapies	1.34 (1.00-1.81)	.0506
Liver metastasis present	1.41 (1.01-1.99)	.0465
Lung metastasis present	1.38 (1.02-1.87)	.0353

Table 2 lists the variables included in our final prognostic score with the hazard ratio (HR) for each individual factor, utilizing multivariate analysis. “Prior therapies” indicates the presence of prior therapies. The HRs listed in the table for continuous variables LDH and neutrophil/lymphocyte ratio, is the interquartile range of the HRs.

Abbreviations: ECOG—Eastern Cooperative Oncology Group.

To assess whether these patient groups had different treatment at the EOL, we assessed the patients that died during evaluable follow up. Among the 398 patients in our cohort, 205 patients died including 45/133 (34%) patients in the low-risk group, 63/133 (47%) patients in the medium-risk group, and 97/132 (73%) in the high-risk group. We analyzed EOL outcomes for the 205 patients who died and compared the outcomes between the 3 risk groups as summarized in Table 3. Approximately, one-third of patients (75/205) received their EOL care at hospitals other than VUMC. Of these 75 patients, the majority (69/75) had known timing of checkpoint inhibitor prior to death. However, it was unclear in 45/75 patients if they were referred for hospice enrollment, and it was unknown in 46/75 patients whether they died at home or in the hospital.

**Table 3.** End of life outcomes among prognostic risk groups.

End of life outcome		g			*χ* ^2^	*P*-value
		Low risk (death = 45)	Medium risk (death = 63)	High risk (death = 97)		
Referral to hospice	Yes	29 (78%)	36 (75%)	54 (72%)	0.384	.825
	No	8	12	20		
Location of death	Home	15 (68%)	24 (62%)	38 (64%)	0.273	.873
	Hospital	7	15	21		
Therapy within 14 days of death	Yes	2 (4%)	0 (0%)	9 (9.5%)	6.287	.043
	No	41	59	86		
Therapy within 30 days of death	Yes	6 (14%)	5 (8.5%)	29 (30%)	12.309	.002
	No	37	54	66		
Therapy within 90 days of death	Yes	15 (35%)	20 (34%)	55 (58%)	11.032	.004
	No	28	39	40		

Table 3 lists the end-of-life outcomes for each prognostic risk group along with their corresponding chi squared value and *P*-values. For referral to hospice, data were available for 159/205 patients. For location of death, data were available for 120/205 patients. With regard to timing of immune checkpoint inhibitor therapy relative to death, data were available for 197/205 (96%) of patients who died.

With regard to timing of ICI therapy relative to death, data were available for 197/205 (96%) of patients who died. Within 14 days of death, 2/43 (4%) of low-risk patients, 0/59 (0%) of medium-risk patients, and 9/95 (9.5%) of high-risk patients received immunotherapy (*P* = .043). Similarly, 6/43 (14%) low-risk patients, 5/59 (8.5%) medium-risk patients, and 29/95 (30%) of high-risk patients received immunotherapy within 30 days of death (*P* = .002). Finally, 15/43 (35%) low-risk patients, 20/59 (34%) medium-risk patients, and 55/95 (58%) high-risk patients received immunotherapy within 90 days of death (*P* = .004).

In terms of referrals to hospice for those patients who died, data were available for 159/205 patients. In this subset, 29/37 (78%) low-risk patients, 36/48 (75%) medium-risk patients, and 54/74 (72%) high-risk patients were referred to hospice (*P* = .825). For location of death, data were available for 120/205 patients: 15/22 (68%) low-risk patients, 24/39 (62%) medium-risk patients, and 38/59 (64%) of high-risk patients died at home (*P* = .873). We detected no differences in proportions of hospice referrals or location of death based on risk strata.

## Discussion

Use of cytotoxic chemotherapy near the EOL is a well-established measure of low-value care, as it causes increased toxicity and costs, worsens quality of life, and has minimal if any clinical benefit.^11,18^ However, the favorable side effect profile and potential benefit of ICIs has made for more challenging EOL decisions and reticence to withhold treatment. As a result, several studies have suggested an overall increase in systemic therapy usage at the EOL, potentially leading to decreased or delayed hospice enrollment, increased inpatient mortality, and increased cost.^10,11,19^ Moreover, providing systemic therapy near the EOL may be discordant with patients’ values and may impede patients from achieving closure and spending their remaining time with loved ones. Therefore, we studied EOL outcomes and current practice patterns at our institution for subsets of patients with metastatic melanoma treated with ICIs who may be at highest risk of mortality to guide future intervention and optimize care.

In this study, we confirmed predictors to identify and stratify patients with advanced melanoma into prognostic subgroups. This prognostic model is driven by known, routinely obtained clinical and laboratory variables that portend poor survival outcomes, including LDH, neutrophil/lymphocyte ratio, prior therapies, the presence of lung or liver metastases, and ECOG performance status. Our study adds to existing literature by subsequently juxtaposing and analyzing prognostic strata with EOL outcomes. Within these risk groups, there was a striking difference in the rates of patients receiving ICI within 14, 30, and 90 days of death, with a greater proportion of patients in the high-risk group receiving therapy closer to death compared to those in medium- and low-risk groups. However, we did not observe differences in patterns of EOL care among these groups, with similar rates of hospice referrals and location of death, even among patients with the poorest initial prognosis. In fact, the proportion of patients who were referred for hospice enrollment and who died at home remained high across prognostic groups, regardless of whether these patients received ICIs near the EOL. Thus, our results reveal an inconsistency in practice patterns between providing ICIs near the EOL and a simultaneous acknowledgement of comfort-focused care in patients with advanced melanoma.

One explanation for the greater proportion of high-risk patients receiving ICIs near death, as compared to those patients in medium- and low-risk groups, is the possibility of decompensation due to irAEs, either due to severe irAEs, or mild-to-moderate irAEs in patients with poor baseline performance status. Although we cannot prove causality in this analysis, prior data have shown that most hospitalizations for patients with melanoma on ICIs are still due to disease and not from irAEs.^20^ Of note, for those with irAEs, overall fatality rates remain low, but greater fatal toxicity was observed early after combined treatment initiation and causes of irAE-related death varied based on therapeutic regimen.^21^ As approximately one-third of the patients in our cohort received combination ICI, it is, therefore, worth noting that for patients at higher risk and with poorer performance status, ICIs could have increased morbidity and hastened death.

With that said, it is critically important to highlight the potential benefit for ICIs in this patient population, which adds further complexity when making therapeutic decisions, even for the highest risk prognostic groups. Our study demonstrates that 27% of high-risk patients in our cohort did not die, and approximately 34% of high-risk patients survived over 1 year from ICI therapy, with most patients receiving first-line therapy. Thus, given the inherent uncertainty of response to treatment in patients with poorer prognoses, our study does not suggest denying patients the opportunity to receive ICIs; it instead emphasizes that potentially beneficial cancer treatment should be balanced with improved efforts for comanagement with integrated palliative care.

Identifying prognostic subgroups could be used by oncology care teams for personalized preparation for EOL care and to improve selective ICI utilization. For example, targeted use of early consultation with palliative care specialists in poor prognosis patients might facilitate more effective care transitions at EOL and limit ineffective, low value care in this setting. Interventions could focus on multiple factors that have been shown to have a positive impact on EOL care including ensuring patients have a clear understanding of their illness, treatment options, and prognostic awareness. An understanding of these factors can help patients plan accordingly for their future and make decisions that are more aligned with their goals and desires.^22^ Furthermore, these conversations have been shown to be associated with greater use of hospice and less aggressive care at the EOL.^23,24^ Although it is recommended to incorporate early palliative care for all patients with cancer, the exact method to do so can be challenging and not-sustainable due to resource limitations that are unique to each hospital system. A tiered approach is more favorable, as it encourages oncologists and oncology nurse professionals to provide supportive care early on, while palliative care specialists can help address more complex needs as they arise.

Our prognostic risk assessment needs further validation to assess how it may ultimately influence oncologist behavior and decision-making, especially with regard to providing ICIs. However, this analysis helps provide a blueprint for identifying patients who could benefit from early palliative care comanagement. A patient’s expected prognosis could be leveraged to better inform and optimize serious illness conversations, and thereby, could influence oncologic decisions about initiating and continuing ICIs. Such nuanced and iterative discussions that focus on patients’ wishes for their care and understanding of prognosis, along with the potential for disease response, should be conducted by primary oncologists and at times, with the added expertise from palliative care specialists.

This study has several limitations. First, when evaluating EOL outcomes, there were many unknown data points especially with regard to location of death, hospice referral, and events immediately around the time of death, as many patients received urgent EOL care at hospitals other than our institution. Our single center analysis also limits generalizability. While our prognostic model focused on clinical and laboratory variables that have proven implications for patients with melanoma, our model did not account for non-disease-specific factors, such as comorbidities, cancer-related symptoms, cancer malnutrition, or cachexia, that could have each impacted survival outcomes prior to starting ICIs. In addition, the results of our study are specific to patients with metastatic melanoma receiving ICIs and are not generalizable to other malignancies that also utilize ICIs. Future studies will require disease-specific prognostic analyses to evaluate associations with EOL outcomes. Finally, the retrospective nature of our study did not differentiate how management of these patients evolved over the 11-year period included in our analysis. For example, oncologists’ experience and practice patterns in utilizing ICIs might have changed over time, as it relates to initiation of ICIs relative to the EOL.

### Conclusion

The implications of our study are notable, as it shows that patients with metastatic melanoma at highest risk of death are more likely to receive ICI therapy closer to death. These patients should be considered for earlier palliative care comanagement strategies to facilitate treatment decision-making, to inform serious-illness conversations, and to ensure that personal goals, and values are honored at the end of life.

## Data Availability

The data underlying this article will be shared on reasonable request to the corresponding author.

## References

[1] Siegel RL , Miller KD, Fuchs HE, Jemal A. Cancer statistics, 2021. CA Cancer J Clin. 2021;71(1):7-33. 10.3322/caac.21654. 33433946

[2] Korn EL , Liu P-Y, Lee SJ, et al. Meta-analysis of phase II cooperative group trials in metastatic stage IV melanoma to determine ­progression-free and overall survival benchmarks for future phase II trials. J Clin Oncol. 2008;26(4):527-534. 10.1200/JCO.2007.12.7837. 18235113

[3] Davis LE , Shalin SC, Tackett AJ. Current state of melanoma diagnosis and treatment. Cancer Biol Ther. 2019;20(11):1366-1379. 10.1080/15384047.2019.1640032. 31366280 PMC6804807

[4] Carlino MS , Larkin J, Long GV. Immune checkpoint inhibitors in melanoma. Lancet. 2021;398(10304):1002-1014. 10.1016/S0140-6736(21)01206-X. 34509219

[5] Larkin J , Chiarion-Sileni V, Gonzalez R, et al. Combined nivolumab and ipilimumab or monotherapy in untreated melanoma. N Engl J Med. 2015;373(1):23-34. 10.1056/NEJMoa1504030. 26027431 PMC5698905

[6] Luke JJ , Flaherty KT, Ribas A, Long GV. Targeted agents and immunotherapies: optimizing outcomes in melanoma. Nat Rev Clin Oncol. Aug 2017;14(8):463-482. 10.1038/nrclinonc.2017.43. 28374786

[7] Wolchok JD , Chiarion-Sileni V, Gonzalez R, et al. Overall survival with combined nivolumab and ipilimumab in advanced melanoma. N Engl J Med. 2017;377(14):1345-1356. 10.1056/NEJMoa1709684. 28889792 PMC5706778

[8] Riaz F , Gan G, Li F, et al. Adoption of immune checkpoint inhibitors and patterns of care at the end of life. JCO Oncol Pract. 2020;16(11):e1355-e1370. 10.1200/OP.20.00010. 32678688 PMC8189605

[9] Parikh RB , Galsky MD, Gyawali B, et al. Trends in checkpoint inhibitor therapy for advanced urothelial cell carcinoma at the end of life: insights from real-world practice. Oncologist. 2019;24(6):e397-e399. 10.1634/theoncologist.2019-0039. 30944183 PMC6656487

[10] Khaki AR , Li A, Diamantopoulos LN, et al. Impact of performance status on treatment outcomes: a real-world study of advanced urothelial cancer treated with immune checkpoint inhibitors. Cancer. 2020;126(6):1208-1216. 10.1002/cncr.32645. 31829450 PMC7050422

[11] Petrillo LA , El-Jawahri A, Nipp RD, et al. Performance status and end-of-life care among adults with non-small cell lung cancer receiving immune checkpoint inhibitors. Cancer. 2020;126(10):2288-2295. 10.1002/cncr.32782. 32142165

[12] Pires da Silva I , Ahmed T, McQuade JL, et al. Clinical models to define response and survival with anti-pd-1 antibodies alone or combined with ipilimumab in metastatic melanoma. J Clin Oncol. Apr 1 2022;40(10):1068-1080. 10.1200/JCO.21.01701. 35143285

[13] van Breeschoten J , Ismail RK, Wouters M, et al. End-of-life use of systemic therapy in patients with advanced melanoma: a nationwide cohort study. JCO Oncol Pract. 2022;18(10):e1611-e1620. 10.1200/op.22.00061. 35944229

[14] Wolchok JD , Chiarion-Sileni V, Gonzalez R, et al. Overall survival with combined nivolumab and ipilimumab in advanced melanoma. N Engl J Med. 2017;377(14):1345-1356. 10.1056/NEJMoa1709684. 28889792 PMC5706778

[15] Ribas A , Hamid O, Daud A, et al. Association of pembrolizumab with tumor response and survival among patients with advanced melanoma. JAMA. 2016;315(15):1600-1609. 10.1001/jama.2016.4059. 27092830

[16] Zhang Y , Liu B, Kotenko S, Li W. Prognostic value of neutrophil-lymphocyte ratio and lactate dehydrogenase in melanoma patients treated with immune checkpoint inhibitors: A systematic review and meta-analysis. Medicine (Baltim). 2022;101(32):e29536. 10.1097/MD.0000000000029536 PMC9371534 35960066

[17] Swetter SM , Thompson JA, Albertini MR, et al. NCCN Guidelines® insights: melanoma: cutaneous, Version 2.2021. J Natl Compr Canc Netw. 2021;19(4):364-376. 10.6004/jnccn.2021.0018. 33845460

[18] Prigerson HG , Bao Y, Shah MA, et al. Chemotherapy use, performance status, and quality of life at the end of life. JAMA Oncol. 2015;1(6):778-784. 10.1001/jamaoncol.2015.2378. 26203912 PMC4828728

[19] Glisch C , Saeidzadeh S, Snyders T, et al. Immune checkpoint inhibitor use near the end of life: a single-center retrospective study. J Palliat Med. 2020;23(7):977-979. 10.1089/jpm.2019.0383. 31702481

[20] Wang LX , Quach HT, Moodabigil NV, et al. Health care utilization and steroid-refractory toxicities from immune checkpoint inhibitors. Cancer. 2020;126(2):322-328. 10.1002/cncr.32542. 31580492 PMC6952563

[21] Wang DY , Salem JE, Cohen JV, et al. Fatal toxic effects associated with immune checkpoint inhibitors: a systematic review and meta-analysis. JAMA Oncol. 2018;4(12):1721-1728. 10.1001/jamaoncol.2018.3923. 30242316 PMC6440712

[22] Kiely BE , Stockler MR, Tattersall MH. Thinking and talking about life expectancy in incurable cancer. Semin Oncol. 2011;38(3):380-385. 10.1053/j.seminoncol.2011.03.007. 21600367

[23] Mack JW , Cronin A, Keating NL, et al. Associations between end-of-life discussion characteristics and care received near death: a prospective cohort study. J Clin Oncol. 2012;30(35):4387-4395. 10.1200/JCO.2012.43.6055. 23150700 PMC3675701

[24] Wright AA , Zhang B, Ray A, et al. Associations between end-of-life discussions, patient mental health. Medical Care Near Death, and Caregiver Bereavement Adjustment. JAMA. 2008;300(14):1665-1673. 10.1001/jama.300.14.1665. PMC2853806 18840840

